# A survey on clinical natural language processing in the United Kingdom from 2007 to 2022

**DOI:** 10.1038/s41746-022-00730-6

**Published:** 2022-12-21

**Authors:** Honghan Wu, Minhong Wang, Jinge Wu, Farah Francis, Yun-Hsuan Chang, Alex Shavick, Hang Dong, Michael T. C. Poon, Natalie Fitzpatrick, Adam P. Levine, Luke T. Slater, Alex Handy, Andreas Karwath, Georgios V. Gkoutos, Claude Chelala, Anoop Dinesh Shah, Robert Stewart, Nigel Collier, Beatrice Alex, William Whiteley, Cathie Sudlow, Angus Roberts, Richard J. B. Dobson

**Affiliations:** 1 Institute of Health Informatics, University College London, London, UK; 2 Usher Institute, University of Edinburgh, Edinburgh, UK; 3 Research Department of Pathology, UCL Cancer Institute, University College London, London, UK; 4 Department of Computer Science, University of Oxford, Oxford, UK; 5 Institute of Cancer and Genomics, University of Birmingham, Birmingham, UK; 6 University College London Hospitals NHS Trust, London, UK; 7 Centre for Tumour Biology, Barts Cancer Institute, Queen Mary University of London, London, UK; 8 Department of Psychological Medicine, Institute of Psychiatry, Psychology and Neuroscience (IoPPN), King’s College London, London, UK; 9 South London and Maudsley NHS Foundation Trust, London, UK; 10 Theoretical and Applied Linguistics, Faculty of Modern & Medieval Languages & Linguistics, University of Cambridge, Cambridge, UK; 11 Edinburgh Futures Institute, University of Edinburgh, Edinburgh, UK; 12 Department of Biostatistics & Health Informatics, King’s College London, London, UK

**Keywords:** Computational science, Translational research

## Abstract

Much of the knowledge and information needed for enabling high-quality clinical research is stored in free-text format. Natural language processing (NLP) has been used to extract information from these sources at scale for several decades. This paper aims to present a comprehensive review of clinical NLP for the past 15 years in the UK to identify the community, depict its evolution, analyse methodologies and applications, and identify the main barriers. We collect a dataset of clinical NLP projects (*n* = 94; *£* = 41.97 m) funded by UK funders or the European Union’s funding programmes. Additionally, we extract details on 9 funders, 137 organisations, 139 persons and 431 research papers. Networks are created from timestamped data interlinking all entities, and network analysis is subsequently applied to generate insights. 431 publications are identified as part of a literature review, of which 107 are eligible for final analysis. Results show, not surprisingly, clinical NLP in the UK has increased substantially in the last 15 years: the total budget in the period of 2019–2022 was 80 times that of 2007–2010. However, the effort is required to deepen areas such as disease (sub-)phenotyping and broaden application domains. There is also a need to improve links between academia and industry and enable deployments in real-world settings for the realisation of clinical NLP’s great potential in care delivery. The major barriers include research and development access to hospital data, lack of capable computational resources in the right places, the scarcity of labelled data and barriers to sharing of pretrained models.

## Introduction

Free-text components of Electronic Health Records (EHRs) contain much of the valuable information that is essential to facilitate tailored care and personalised treatments for patients^1–3^. A lot of this information is either unlikely to be available or is more comprehensive than the structured component of EHRs only^4,5^. Data such as signs or symptoms of disease, adverse drug reactions, lifestyle (e.g. smoking, alcohol consumption and living arrangements), family medical history, or key information describing disease subtypes are recorded with greater frequency and depth in free-text data^6–8^. To interrogate free texts and unlock deep phenotypic data for research and care, Natural Language Processing (NLP) approaches^2–4,6–8^ have been adopted to automate the extraction of such information at scale. Like any NLP task, clinical NLP needs to tackle the challenges of devising computer programmes for understanding human spoken or written languages, which constitute some of the most challenging problems faced by artificial intelligence (AI). For those implementing or using clinical NLP, there are additional complications and challenges, which, on the flip side, are new opportunities for research and development.

Clinical NLP often encounters challenges with insufficient data for both supervised and unsupervised machine learning (ML). This ‘low-resource’ setting can be considered in three contexts. First, labelled data for supervised models are scarce and ‘expensive’; these are difficult to scale. Annotators require medical expertise to evaluate clinical information to generate ground truth. Disagreements are prevalent and long-standing among clinical experts^9–11^. The annotation process often requires multiple clinician annotators with senior clinicians, who often have other clinical commitments, adjudicating disagreements. Second, clinical NLP tasks are very likely to deal with highly imbalanced data, which is widely perceived as challenging for ML algorithms^12^. For example, an NLP study examining radiology reports of brain scans^13^ reported the most frequent phenotype as *Ischaemic Stroke* (*n* = 2706 or 11.6%) and the least frequent as *Meningioma Tumour* (*n* = 10 or 0.4%). The third ‘low resource’ is a computational resource. NLP systems often require capable computational environments with software such as Python, libraries, or open source repositories and hardware, including graphic processing units. It is technically challenging to set up these computational requirements in trusted research environments (TREs), such as those within hospital networks, where clinical data is securely accessible.

Clinical NLP is also knowledge-intensive—the need to incorporate formalised knowledge that computers can understand. Domain knowledge has been shown to be important for understanding biomedical texts, such as in interpreting linguistic structures^14^. Medical text report classifications were also shown to benefit significantly from expert knowledge^15^. In terms of knowledge-based computation, a common feature of clinical NLP applications is the need to perform patient-level inferences, in addition to standard tasks, such as identification of named entities or document classification; an example of this is the inference of subtypes of stroke based on named entities retrieved from text reports^8^.

The knowledge, commonly represented as ontologies, required for clinical decision-making falls at the intersection of many biomedical sciences, including epidemiology, genetics, pharmacology and diagnostics. The size and breadth of background knowledge needed to make inferences are great. However, clinical NLP benefits from the availability of massive knowledge resources that support biomedical science. Medical vocabularies such as SNOMED CT^16^ and ICD-10^17^ provide classifications of clinical concepts that include taxonomy and vocabulary. In addition to these features, biomedical ontologies provide a formal semantics for a wide range of biomedical concepts and their inter-relations^18,19^. Despite the development of these knowledge resources, clinical knowledge at the patient level is largely not represented in a computer-usable form; for example, no existing ontologies can inform an AI system that, while possible^20^, it is probably inconsistent to diagnose a patient with both types 1 and type 2 diabetes. Developing formal knowledge resources is a current challenge for enabling and improving clinical decision-making applications.

Lastly, access to patient-level free-text clinical data is controlled by information governance (IG) regulations^21^, such as the UK’s legal framework^22^, including the NHS Act 2006, the Health and Social Care Act 2012, the Data Protection Act and the Human Rights Act. These regulations are usually complex. The interpretation and application are varied, often resulting in defensive practices. For example, while it is widely acknowledged that it is difficult to comprehensively anonymize free-text data, there is much less consensus on how to do text anonymization at scale, what are the proper evaluation procedures, what level of performance is good enough, and how the anonymization fits within a framework that would ensure confidentiality according to the regulations. As a result, data access to patient data is one of the biggest hurdles for clinical NLP. There has been progress in developing in-house NLP within large NHS organisations such as hospitals, but the IG challenges are greater for using data across NHS organizations.

These challenges (or opportunities) faced by clinical NLP are too great to be tackled by individuals working alone or in small research groups. Cross-organisation collaboration is key to addressing technical challenges, such as sharing data or models, yet the NLP community remains fragmented. Formalising patient-level inference knowledge at scale is only feasible as part of a community effort. Furthermore, national coordination is necessary to create reproducible streamlined procedures for facilitating access to free-text clinical data.

There is a large body of literature reviewing clinical NLP, providing useful summaries of the developments of technologies and applications, for example, on application domains^23,24^, on particular clinical questions^25,26^, on particular modalities^27–29^, or on methodologies^30–32^. However, healthcare services and their regulations (e.g., the above-mentioned IG policies) differ from country to country. Clinical NLP would particularly benefit from close collaborations and coordination initiatives at a national level. None of the existing reviews provides a comprehensive overview (including who and what, the developments and the gaps) for facilitating such *national-level* collaborations.

This article aims to facilitate an informed national effort to tackle grand clinical NLP challenges, through a network-based, timestamped and multifaceted review and analysis of the development of clinical NLP in the UK over the past 15 years. Specifically, the main objectives are to gain an understanding of the following key aspects:

- **Who:** To identify the key stakeholders, including organisations (funders, universities, NHS Trusts and companies) and persons (researchers, students and developers) and how they are connected to each other to form the community.
- **What:** To survey the applications, clinical questions, technologies and datasets the community has been working on.
- **Where:** To uncover how the community has grown and how technologies and application domains have evolved over the years; in particular, to assess how the technologies have been used in real-world settings and how the technology maturity levels have changed.
- **Gaps:** Importantly, we identify the gaps that require investment from funders, the barriers to unlocking the potential of clinical NLP and the future research directions.

**Fig. 1:**
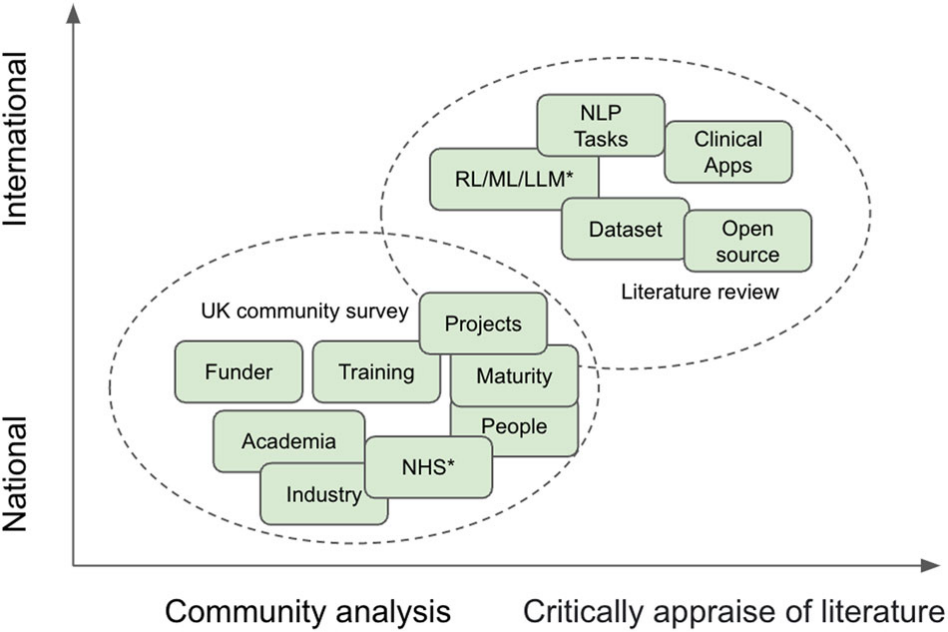
The scope of this study is composed of two main parts. **a** A UK community survey (the lower oval); and **b** a literature review of the community’s research outputs (the upper oval). ^*^NHS—National Health Service in the UK; RL/ML/LLM—NLP technologies of rule-based, machine learning and large language models.

The scope of this study is depicted in Fig. 1 and comprises two parts. The first is to conduct a community analysis of UK clinical NLP in the last 15 years. This is to reveal the key stakeholders, their connections and developments. The second is to conduct a literature review on the research outputs of the community to understand the technologies used, key application domains and their trends.

## Results

### Clinical NLP community analysis results

**Fig. 2:**
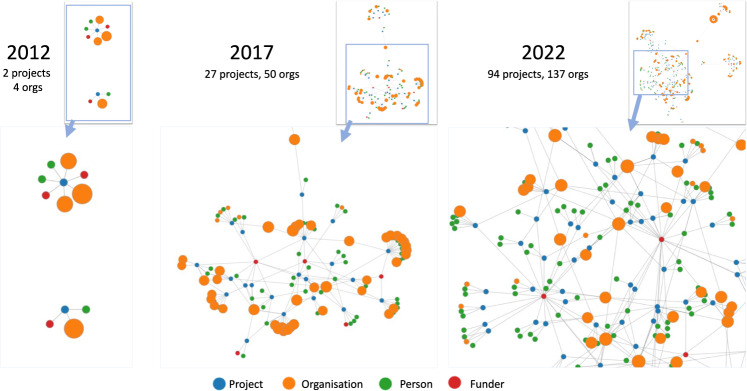
Snapshots (force-directed visualisations) of the community from 2012 to 2022. The graphs contain four types of entities: projects, persons, organisations and funders. Each graph is constructed using data from projects with a start date earlier than or in the given year. Graph data is cumulative, meaning a later year’s data is a superset of its previous years. The size of organisation nodes indicates the number of total amounts in pound sterling they received in funding.

For overall community developments, Fig. 2 illustrates overall graph representations of the clinical NLP landscape at three-time points (five years apart): 2012, 2017 and 2022. It shows a steady trend of rapid and significant developments in the community in the last 10 years. By 2012, there were only two funded projects involving four organisations with a total of £0.37 million funding. Five years later, by 2017, there were 27 projects and 50 organisations with a total funding of £10.35 million. The latest data collected in this study (by February 2022) shows there were 94 projects, 137 organisations and a total funding of £41.97 million. Interactive visualisation of the graph is available at https://observablehq.com/@626e582587f7e383/uk-clinical-nlp-landscaping-analysis#chart.

To identify the key stakeholders in the community, we ranked the nodes in the graph by their relative centrality scores based on the Eigenvector centrality measurement. Table 1 shows the three ranked lists of organisations stratified by type. The first part of the table (Table 1a) lists the top 10 most influential organisations of all types; the second part (Table 1b) lists the top NHS organisations, and Table 1c lists the top 5 industry organisations. The top 10 most influential organisations are all universities. The combined influence (=24.62) of the top 5 universities is more than 3.7 times the sum of the influence of all NHS Trusts in the community and more than 4 times that of the top 5 industry institutions.

**Table 1:** Different types of organisations are ranked by eigenvector centrality scores relative to the median value of those of all organisations.

Organisation name	RCS	Amount (£m)
*Top ten organisations of all types*		
University of Manchester	6.53	6.14
University College London	5.23	3.54
University of Cambridge	4.53	3.76
University of Edinburgh	4.35	6.31
Imperial College London	3.98	1.55
King’s College London	3.97	3.56
University of Oxford	3.3	0.72
University of Liverpool	3.27	2.61
Lancaster University	3.25	1.19
University of York	2.11	1.45
*Top NHS organisations*		
Salford Royal NHS Foundation Trust	1.53	1.28
NHS Greater Glasgow and Clyde	1.05	0.05
University College London Hospitals NHS Foundation Trust	1.03	0.64
Berkshire Healthcare NHS Foundation Trust	1.03	0.97
Nottinghamshire Healthcare NHS Foundation Trust	1.03	0.37
South London and Maudsley (SLAM) NHS Foundation Trust	0.87	0.31
*Top industry organisations*		
Abtrace Limited	1.58	2.19
FACTMATA LIMITED	1.11	0.06
MENDELIAN LTD	1.11	0.10
Mantrah Limited	1.06	0.14
Swifter Limited	1.06	0.13

*RCS* relative centrality score.

Most NHS and industry organisations have a relative centrality score larger than one (i.e., higher than the median centrality score), meaning they are involved in relatively highly influential projects.

**Fig. 3:**
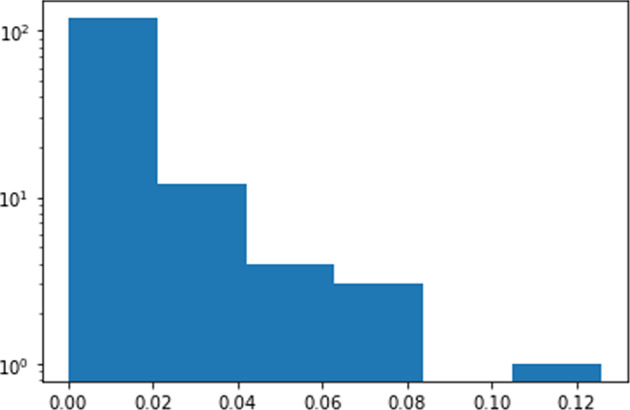
Histogram of person nodes Eigenvector centrality scores. The *x*-axis is the eigenvector centrality score and the *y*-axis (log scale) is the number of people with certain scores.

For individuals, Fig. 3 illustrates the histogram of absolute eigenvector scores of all persons in the community. It shows a likely long-trail distribution.

To reflect on technology take-ups and maturity, we did an analysis of the involvement of industry partners and deployment within health services, both of which are key indicators for the maturity of a technology.

**Fig. 4:**
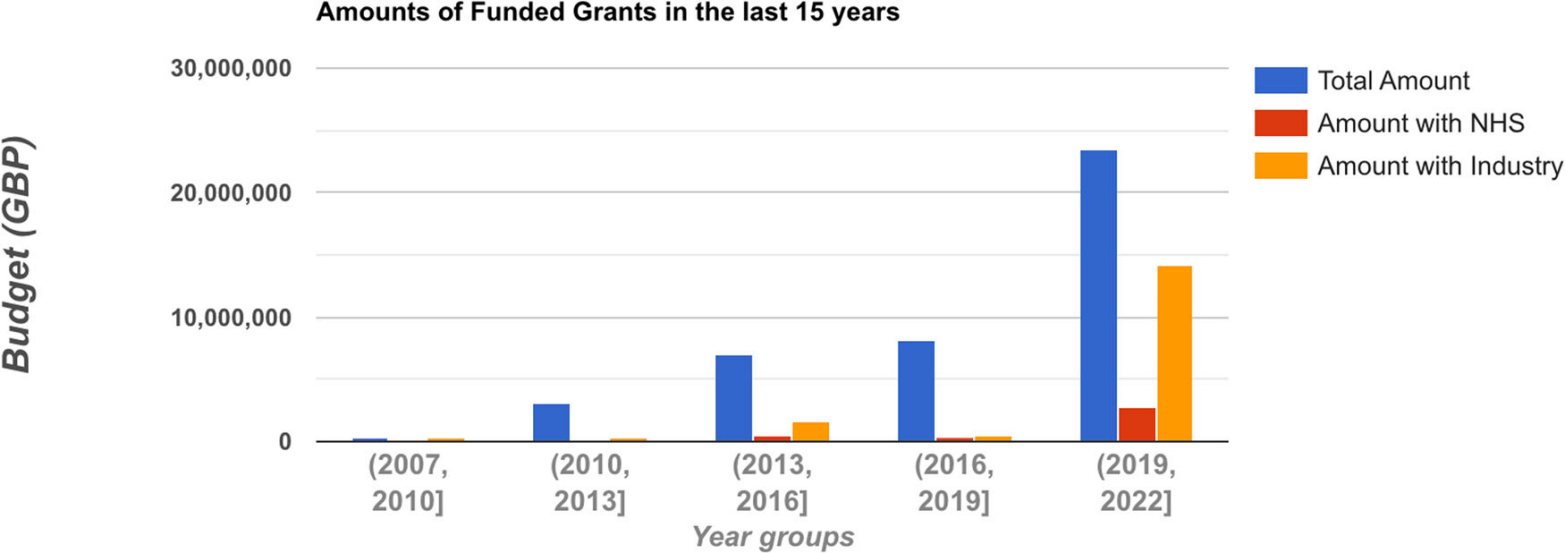
Trends in the last 15 years on budgets of all clinical NLP projects, those involving NHS and those involving industry organisations. Each tick on the x-axis is a 3-year period. The y-axis shows the total budget. The sums of NHS involved and industry involved project budgets are plotted alongside the budget of all projects across five 3-year periods.

Figure 4 shows the budget trends for all projects, projects that involved the NHS and projects that involved industry in the last 15 years grouped by 3-year periods. It shows a clear pattern whereby the funding for clinical NLP in all three categories has increased significantly. It is particularly encouraging to see NHS organisations’ involvement in this area has markedly increased in the last three years. Industry involvement has increased more than 27 times from the 2016-2019 period to the 2019–2022 period.

To understand the interactions between groups in the community, it is important to know: (1) what the key subgroups are; and (2) how they are connected with each other.

From the 2022 snapshot in Fig. 2, we observe that there are four natural clusters in the graph. The middle of the graph is the biggest cluster, containing research projects supported by UK research councils such as EPSC, MRC, BBSRC and ESRC. The top left corner forms the second cluster, which is NIHR-funded projects. The NIHR funds health and social care research, which is supposed to be more translational than research in the main cluster. The third cluster is on the right and contains projects funded by Innovate UK. Such projects are sometimes led by industry and are intended to produce products ready for use by end customers, i.e., health service providers such as the NHS. The top right is the cluster of projects funded by EU Horizon 2020 (H2020) programmes. Overall, the four clusters are connected weekly with each other.

To quantify the strength of connections between subgroups within the community, we conducted a *k*-connectivity community analysis. Table 2 shows the results, where a sub-community is represented by a funder composed of its funded projects and associated persons and organisations. The community is connected. Therefore, when k is 1, the whole graph constitutes one and only one connected component. When *k* = 2, Innovative UK and H2020 sub-communities are separated from the main component. When *k* = 3, the whole subgraph of Innovative UK disappears, meaning the connectivity within its own cluster is also weak. The same applies to H2020 projects.

**Table 2:** K-connectivity analysis results on the network. A funder name represents the sub-community composed of the funder, its funded projects and associated persons and organisations.

*k* values	Component 1	Component 2	Component 3
1	〈WHOLE GRAPH〉		
2	NIHR; BBSRC; EPSRC; MRC; ESRC	Innovate UK	H2020
3–4	NIHR; BBSRC; EPSRC; MRC; ESRC		
5	NIHR; EPSRC; MRC; ESRC		
6–8	EPSRC; NIHR; MRC		
9–16	EPSRC; MRC		

*NIHR* National Institute for Health Research, *BBSRC* Biotechnology and Biological Sciences Research Council, *EPSRC* Engineering and Physical Sciences Research Council, *MRC* Medical Research Council, *ESRC* Economic and Social Research Council.

For the main cluster where all other funders reside, the connectivity is not strong: BBSRC disconnected at *k* = 5, ESRC disconnected at 6 and NIHR disconnected at 9. EPSRC and MRC form the core, which keeps inter-connected until k reaches 17.

It is worth mentioning that as of 1st January 2020, the graph of the whole community was composed of three separate components of H2020, Innovative UK and other funders. This means the community was formed as an interlinked graph for just a little more than two years.

**Fig. 5:**
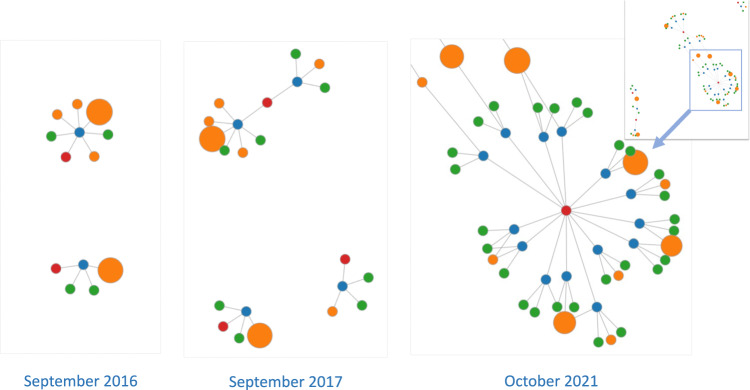
The development of studentship projects in clinical NLP from 2016 to 2021. The three figures (from left to right) show the networks of studentship projects and their associated entities (funders, organisations and persons) for 2016, 2017 and 2021 respectively. The 2021 entire network is too big to be shown fully using the same scale. Therefore, a low-resolution overview is shown at the top right and a snapshot of it is displayed using the same scale as other years.

For depicting the development of training next-generation clinical NLP Leaders, we extracted studentship projects (i.e., funded via doctoral training programmes) to understand the trends of clinical NLP-related PhD projects in recent years. Figure 5 shows three snapshots of funded studentship projects in 2016, 2017 and 2021, respectively. The first project was funded by the MRC, led by Edinburgh and started in 2016. By October 2021, there were a total of 16 funded studentship projects identified, out of which 10 were funded by EPSRC and 5 by MRC.

### Literature review results on publications

A total of 431 publications were extracted from the 94 projects identified in the community analysis above. A manual screening process was conducted using study criteria detailed in the method section, which identified 107 publications for review.

Table 3 lists the key characteristics of the 107 studies in the last 15 years, including 16 published during 2007–2012^33–48^; 31 in 2012–2017^49–79^; and 60 published in 2017–2022^80–139^. More than 45% (*n* = 49) of these studies were international (involving at least one collaborator from a country other than the UK). There were a total of 23 collaborating countries or regions, with Japan (*n* = 12), the USA (*n* = 12) and Sweden (*n* = 11) being the top three most frequent collaborating countries.

**Table 3:** Key characteristics of the included studies (*n* = 107).

Study characteristics	*n* (%)
*Publication year*	
2007–2012	16 (15.0)
2012–2017	31 (29.0)
2017–2022	60 (56.1)
*Country/region of collaboration*	
Japan	12 (11.2)
United States of America	12 (11.2)
Sweden	11 (10.3)
China	8 (7.5)
Australia	5 (4.7)
Italy	3 (2.8)
Others	22 (20.6)
*Natural language processing tasks*	
Named entity recognition	34 (31.8)
Text classification	29 (27.1)
Relation extraction	18 (16.8)
Information retrieval	14 (13.1)
Entity normalisation	3 (2.8)
Temporal expressions	3 (2.8)
Natural language generation	1 (0.9)
Other information extraction	6 (5.6)
*Health category*	
Mental health	23 (21.5)
Treatments	10 (9.3)
Oncology	7 (6.5)
Cardiovascular	4 (3.7)
Infectious	6 (5.6)
Respiratory	5 (4.7)
Autoimmune	1 (0.9)
COVID-19^a^	4 (3.7)
General applicability	60 (56.1)
*Deployment in health services*	
No	101 (94.4)
Deployed in NHS env	6 (5.6)

^a^The COVID-19 category was added in addition to the categories defined at https://www.cdisc.org/standards/therapeutic-areas/disease-area.

Categorised by NLP tasks,

- 31.8% (*n* = 34) performed *named entity recognition* including extractions of phenotypic information^42,55,65,66,85,87,104^, diseases ^50,56,84,89,133^, drug entities^53,95,115^, proteins or genes^39,68,107^ and general concept extractions^52,74,76,81,82,88,90,92,96,103,109,113,122,124,131,137^.
- 27.1% (*n* = 29) performed *text/document classification*, including risk assessment classifications^34,48,49,91,97,99^, literature review^57,114,117,119,120^, drug-related^58,100,116,118^, randomised clinical trials^127–129^ and generic classifications (such as classifying or clustering documents)^51,54,60,75,79,80,105,106,112,135,139^.
- 16.8% (*n* = 18) performed *relation extraction* including event extractions^35,37,59,71^, adverse drug reactions^64,67,69^ and generic information extractions^36,38,40,61,62,70,108,110,121,125,136^.
- 13.1% (*n* = 14) did *Information retrieval* including retrieval from EHR^83,93,94,98,101,102,134,140^, literature data^47,111^ and other types of data^43–45,72,138^.
- Other types of tasks performed included *entity normalisations*^77,78,126^, *temporal expressions*^86,93,134^ and *natural language generation*^63^.

Contextual mentions of phenotypes and diseases are particularly essential in clinical applications. Identifying positive and negated mentions such as the *patient has/has not got fever* is among the most studied contextual named entity recognitions^72,81,98^.

In terms of health categories, mental health was the most widely studied area^84,86–103,132–134^. It was followed by treatment ^53,58,78,79,108,115,116,118,123^, among which drug-related (mostly adverse drug reactions) studies^53,58,115,118^ were most common. Oncology ^33,34,48,49,55,75,117^ and cardiovascular diseases^62,65,66,83^ were the next two most frequently studied areas following treatments. Other disease areas included infectious^42,43,82,96,133,135^, respiratory^56,82,96,133,135^ and autoimmune^138^ diseases. In particular, there were four studies on COVID-19^82,96,133,135^. The rest were studies that belong to the ‘general applicability’ category, meaning they were tools or models not designed for specific health categories or diseases. They have general utilities for particular scenarios that might be applicable to a wide range of clinical use cases^50–52,54,57,59–61,63,74,77,81,85,104–106,109–114,119–122,124,125,131,136,137^.

Of the 107 reviewed papers, 21 (19.6%) of them provided open access to their repositories, making them usable tools/software for the community. As for utilities in real clinical settings, only 5.6% (*n* = 6) studies were deployed or further developed on systems deployed in NHS environments^81–85,140^, of which^81,140^ have been deployed as generic information retrieval or extraction platforms on near real-time EHRs of respective NHS Trusts. Compared to other work, these deployed tools are all concept-linking tools for identifying a broad range of biomedical concepts using large terminologies, including SNOMED CT and UMLS. This makes them suitable for creating a generic platform that can support a wide range of disease areas and application domains.

We conducted further analysis to understand technical objectives vs. NLP tasks. To investigate the clinical application categories, we adapted the classification system from^28^ and made slight changes to classify the studies into the following five technical objectives:

- *Disease information and classification*. This is to use NLP for classifying a disease occurrence or extracting information about a disease with no focus on the overall clinical application. Studies in this category include^34–40,45–49,56,58–62,65–73,76–80,100,103,112,115–118,121,122,124,128–130,134,135,139^.
- *Language discovery and knowledge*. This category studies how ontologies and lexicons could be combined with other NLP methods to represent knowledge that can support clinicians. Studies include^33,41,50–52,55,63,74,75,81,85,86,104–108,110,114,119,131,132^.
- *Diagnostic surveillance*. This is to use NLP for extracting disease information for the patient or disease surveillance^64,82–84,87–93,99,102,125,126,133,136,138^.
- *Cohort building for epidemiological studies*. The objective of this category is to create cohorts for research purposes or support the derivation or analysis of the outcomes of epidemiological analysis. Studies belonging to this category include^57,94–98,101,111,113,120^.
- *Technical NLP*. Other studies include those mainly focused on the technical aspects of NLP, i.e., developing or applying NLP technologies for improving the understanding of clinical free-text. Nine studies belong to this category^42–44,53,54,109,123,127,137^.

Advances in the above three technical objectives in particular (*Disease information and classification, Diagnostic Surveillance and Cohort Building for Epidemiological Studies*) offer a great opportunity for health systems to harness data from unstructured EHRs for better care. In addition, clinical NLP has great potential in (semi-)automated clinical coding for timely and more accurate auditing, surveillance and public health policing^141^. However, at the writing of this review, developments of automated coding are still in their infancy in the UK.

**Fig. 6:**
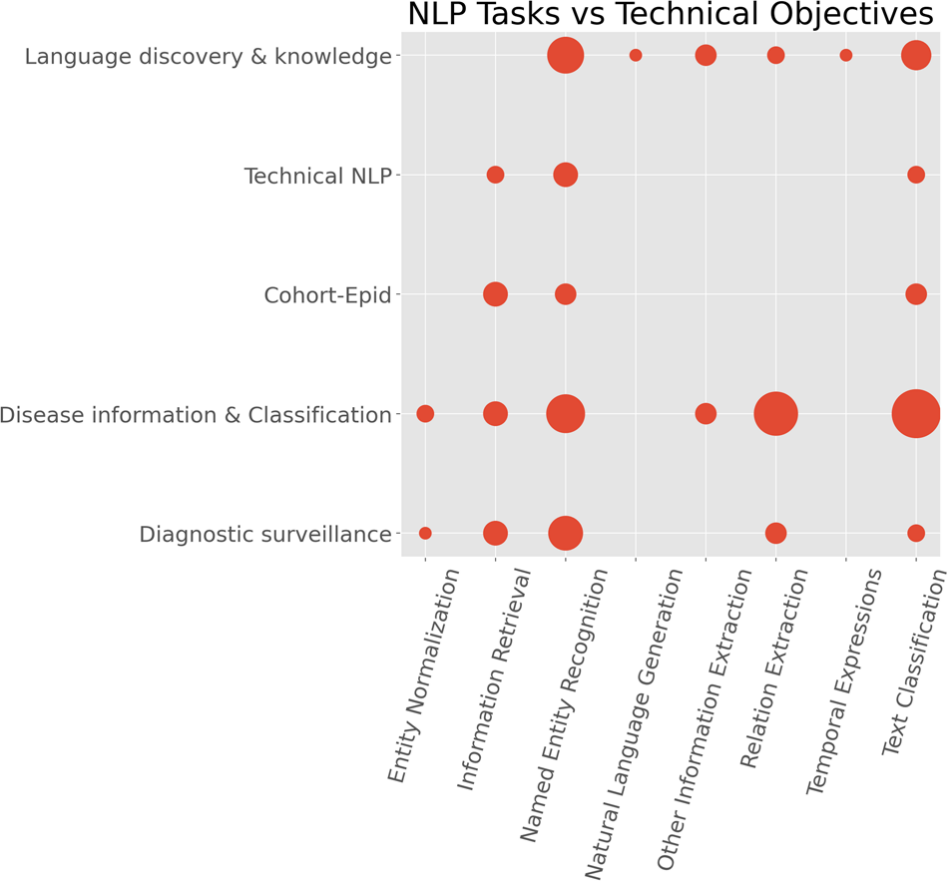
NLP tasks versus technical objectives. The *x*-axis is the categories of NLP tasks and the *y*-axis is the technical objectives. The size of the circles denotes the number of publications.

Figure 6 illustrates a scatter plot of the NLP tasks against the technical objectives. It shows how different NLP technologies have been adopted to address different clinical questions. The largest combination is *Text Classification* with *Disease information & Classification* containing 16 studies^34,48,49,58,60,79,80,100,112,116–118,128,129,135,139^. *Named Entity Recognition* has been widely used in different clinical applications: 10 studies of *Disease information & Classification*^39,56,65,66,68,76,103,115,122,124^, 9 studies of *Language discovery & knowledge*^50,52,55,74,81,85,104,107,131^ and 8 of *Diagnostic surveillance*^82,84,87–90,92,133^. In particular, there were some areas (represented by small circles in the figure) that were clearly understudied, for example, *Text Classification* for *Diagnostic surveillance*^91,99^, *Entity Normalisation* for *Diagnostic surveillance*^126^ and *Natural Language Generation* in any application domains^63^.

**Fig. 7:**
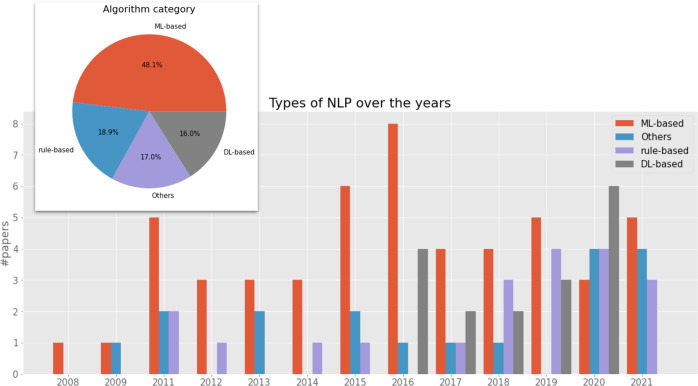
NLP algorithm type breakdown and their development trends over the last 15 years. The main bar chart shows the changes of different NLP algorithms in the last 15 years. The pie chart at the top left corner depicts the overall breakdown of algorithms of all research work analysed.

In terms of NLP technologies and the trend, the pie chart in Fig. 7 summarises the different types of clinical NLP algorithms adopted by the selected 107 studies. When there are multiple algorithm categories, we use the main model or best-performing model’s algorithm type.

*ML-based* denotes those tools using ML algorithms (excluding those using deep neural network methods). There were 48.1% of studies using ML-based methods, including Support Vector Machines^34,45,49,55,80,93,104,127–129^, Bayesian methods^33,34,48,58,72,97^, Conditional Random Fields^33,54,56^, Random Forest^72,93,119^, Logistic Regression^72,93,97^, Artificial Neural Networks^104^, Decision Trees^72^ and others^43,57,78,83–85,113,117,138^.

*Rule-based* describes 18.9% of the studies using manually-created rules for classifications or extractions^37,38,44,53,74,86,94,96,98,99,101–103,105,107,111,115,123,134,135^.

*DL-based* denotes those using deep learning methods, accounting for 16.0% of the studies, including convolutional neural networks^75,77,79,109,110,121,130^, recurrent neural networks^76,77,116,121,122,124^, long short-term memory^76,116,121,122,124^ and transformers^112,116^.

*Others* are those studies where the algorithms were not clearly specified.

The bar chart in the figure shows the development trends of different NLP algorithms used in the community. Traditional ML-based methods peaked around 2015–2016, with DL-based methods becoming increasingly popular thereafter. Rule-based methods started decreasing in 2011 and remained at low-level usage when ML-based methods were popular. Interestingly, they started to increase again in 2018 by both absolute number and percentage.

**Fig. 8:**
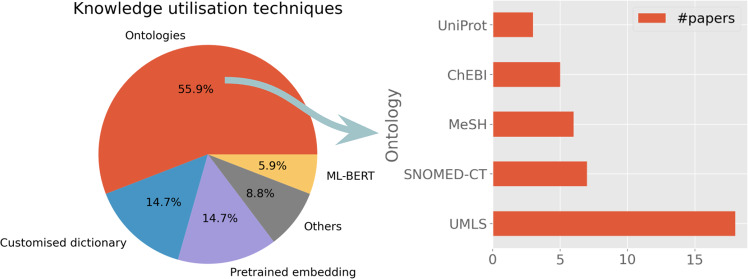
Knowledge representation and distributed representations. The pie chart at the left shows the breakdown of representation techniques. For ontologies, the bar chart on the right depicts the top five frequently used ontologies in clinical NLP applications.

Domain knowledge utilisation is an essential component in many clinical NLP applications. To understand knowledge technologies, we extracted data from the selected studies to analyse how domain-specific knowledge was represented and utilised for facilitating clinical NLP tasks. We defined domain knowledge in a broad sense in this analysis, including both domain-specific ontologies or terminologies (customised dictionaries), distributed representations learned from external corpora (such as dense vector representations of word semantics) and pretrained large language models (e.g., BERT model and its variants). Figure 8 shows the summary of adopted knowledge techniques.

- *Ontologies*: Clinical domain involves a wide range of domain-specific ontologies, from clinical terminologies to biological ontologies to literature classification systems. Overall, 55.9% of studies utilised ontologies, amongst which we identified the five most commonly used ontologies: Unified Medical Language System was used by 16.8% (*n* = 18) studies^36,56,61,64–66,69,70,81,83–85,89,106,113,115,130,139^; SNOMED CT had 6.5% (*n* = 7) users^65,66,78,82,87,115,124^; MeSH was used by 5.6% (*n* = 6) studies^61,67,104,111,115,139^; ChEBI was used by 4.7% (*n* = 5)^56,62,104,113,115^; UniProt 2.8% (*n* = 3) was used by studies (*n* = 3)^56,69,113^.
- *Pretrained embeddings*: Techniques such as word2vec^142^ aim to learn dense vector representations (called embeddings) for words or larger constructs (like phrases) from large external corpora, which capture ‘transferable’ (domain) language semantics for facilitating new tasks. The most used embedding model from the 107 studies was word2vec^76,79,121,122,136^. The second most popular model was FastText^79,121,122,136^. One study^136^ used word2vec, FastText, ElMo, Glove and Flair.
- *Customised dictionary*: Ten studies used customised dictionaries, including cancer studies^55,75^, two drug studies^53,58^, two mental health studies^88,134^, a multilingual study ^51^ and others^105,114,119^.
- *ML-BERT*: Large language models like BERT or their techniques were used by four studies, including a study for identifying cognitive impairments in schizophrenia^100^, event extractions^112^, a social media corpus study^125^ and a pre-trained biomedical entity representation ^137^.
- *Others*: There are studies which adopted hybrid methods, including those using bag-of-words representations^34^, utilising lexical structures^41^, using biological process subontology of the gene ontology (GO)^45^, using multiple methods^77^ including ontologies (SNOMED CT and SIDER) and word2vec, combing UMLS and customised dictionary^50^ and with unspecified methods^63^.

Table 4 summarises the types of datasets used by the studies. The majority of them (54.2%; *n* = 58) used literature corpora^33–40,42–57,59,60,66–76,80,104–107,109,111–115,117,119–121,127–131,139^. Eleven (10.3%) used social media data^58,64,77–79,89,125,126,135,136,138^.

**Table 4:** Dataset sources of studies.

Sources of data	#Studies (%)
Literature	58 (54.2%)
EHR	31 (29.0%)
Social media	11 (10.3%)
Other	7 (6.5%)

In total, 31 (28.97%) studies used real-world EHR data. Amongst them (see Table 5), 21 (67.74%) used South London and Maudsley NHS Foundation Trust mental health Hospital (SLaM) EHR data (CRIS)^81,84–88,90–100,103,132–134^. There were only 4 UK EHRs utilised by the studies. Apart from SLaM, they were from King’s College Hospital (used by ^81–83^), Oxford Health NHS Foundation Trust (OHFT) (used by^101^) and Camden & Islington Trust (used by^102^). None of the UK-based EHRs was openly accessible but were described as being available to collaborators. All three open accessible EHRs were from the US: i2b2 (Informatics for Integrating Biology & the Bedside) (used by^63,65^); n2c2 https://portal.dbmi.hms.harvard.edu/projects/n2c2-nlp/ (used by^116,118^); and MIMIC-III^143^ (used by^81,116,118^. There was one EHR from China—Jinhua People’s Hospital (used by^63^). The largest EHR dataset cited for NLP implementation was CRIS at SLaM, of which reported sizes were 23.3m documents and >400k people. The second largest was OHFT (31,391 people).

**Table 5:** EHR datasets used by studies.

Dataset	#Studies	Region	Open access
SLaM - CRIS	21	UK	
King’s College Hospital	3	UK	
n2c2	2	US	x
i2b2	2	US	x
Jinhua	1	CN	
MIMIC-III	3	US	x
OHFT - Oxford	1	UK	
Camden and Islington	1	UK	

*SLaM* South London and Maudsley Hospital, *OHFT* Oxford Health NHS Foundation Trust, *Jinghua* Jinhua People’s Hospital, *Camden and Islington* Camden and Islington NHS Foundation Trust, *i2b2* https://www.i2b2.org/, *▒n2c2* https://portal.dbmi.hms.harvard.edu/projects/n2c2-nlp/, *▒MIMIC-III* https://physionet.org/content/mimiciii/1.4/.

## Discussion

We conducted a detailed study on clinical NLP developments in the UK for the last 15 years since 2007. A network analysis was conducted on the community dataset, including funders, projects, people, and organisations. A further literature review was carried out to analyse publications from the community. Results from the two analyses revealed multifaceted insights into the evolution of the UK NLP community, and related technical research and developments.

In terms of community developments and connections, clearly, clinical NLP has developed rapidly in the UK. The visualisations of different timestamped snapshots (Fig. 2 shows the community to be steadily expanding over the last 10 years. Analysis of community stakeholders has revealed a consistent power-law distribution of their influences across all types of entities (i.e., funders, organisations, persons and projects). This means that there are ‘key players’ in all types of entities. As for funders, MRC and EPSRC play critical roles. Their funded projects form the core of the community.

For organisations, the dominant influence of universities indicates clinical NLP is still a research-dominated area in the UK. Meanwhile, NHS and industrial organisations have gained considerable influence in the community (see Table 1). These are promising signs that NLP technologies are starting to be taken up by industry and healthcare service providers. Such signs are further confirmed by the analysis of the trends of funding sources that involve these partners. Particularly, industry involvement in projects has increased from less than 1/15 from 2016 to 2019 to around 1/1.5 from 2019 onwards, indicating possible increased technology maturity, or recognition of the potential generally, of clinical NLP in the last 3 years.

Another positive sign observed is the continuously increasing investment in training the next generation of NLP researchers. Since 2016, studentship projects have increased from just one to 16 across 14 institutions. Figure 5 reveals a pattern of continuously increasing studentships overall across different organisations, which is encouraging.

However, links between sub-communities appear to be weak. For example, projects funded by Innovative UK are very weakly linked with other funders and their funded projects/people—only two edges, to be specific. This means the connections between academia and industry sub-communities are fragile. The NIHR and its funded projects, which are supposed to be more translational, also form their own cluster with a similar weak connection to those funded by MRC and EPSRC. Such weak connections might indicate that the translation from research to outputs that directly benefit health services is also weak and not streamlined. These sub-communities mostly work alone. This might indicate barriers to the translation of active research into mature technologies to support business or improve health services.

Our literature review of the 107 selected publications has revealed a strong growth pattern that echoes the expansion of the community from the above network analysis. Specifically, research publications doubled every 5 years in the last 15 years. The community has collaborated with more than 20 countries internationally.

On the aspect of applications and translations, while the studies as a whole have covered a wide range of diseases, the majority were focused on mental health or treatments. The main reason might be that there is a lack of good coverage of coded data in areas such as mental health. For mental health, many symptoms and phenotypes are usually not routinely coded as structured data: for example, the quantification or qualification of cognitive impairment. For treatments (mainly drug-related studies), adverse reactions or events were the main information to be extracted, which are also rarely routinely coded in a structured format. This means current research mainly utilises NLP for uncovering the under-coded information when this is needed across the EHR database as a whole (i.e., in samples too large for manual annotation or checking, and in clinical services where the imposition of structured instruments for routine information-gathering is not feasible or acceptable). The potential of free-text data for subtyping diseases (e.g., revealing the nuance of phenotypic representations) seems less exploited at the current time. This is an area where clinical NLP could maximise its utilities for facilitating personalised medicine as and when in-depth information is demonstrated to have prognostic value.

Regarding technical objectives, the three categories of *language discovery & knowledge*, *disease information & classification* and *technical NLP* combined constitute almost 74% of the studies. This means that only 26% of research-targeted problems are classified as *diagnostic surveillance* and *cohort-epid*, both of which are more clinically actionable. This observation indicates that the current studies are less translational in clinical practice, which reflects the findings from the community analysis. This is also reflected by the very low number (<6%) of deployed clinical NLP systems within NHS environments.

Such a low level of development might reflect the big challenges faced by translation to health systems. Among others, deployments of NLP models on production EHR systems do encounter additional technical challenges. For example, compared to research-oriented NLP, translational model developments would mean moving from relatively small-volume evaluation datasets to applications at scale across very large and diverse corpora, making high generalisability an essential requirement. In addition, these models might encounter a near-inevitable drop-off in performance either from annotation-level to whole-patient-timeline-level evaluation due to the shift of data patterns over time or gradual changes within the clinical practices. Further to this, there is also the challenge of translating the application of NLP across large historic datasets into incorporation pipelines of real-time processing of clinical text within the EHR for individual-level feedback, as well as the utility challenge of communicating probabilistic clinical decision support where NLP models are not 100% accurate, and finding case studies that make use of new capabilities (the ‘solution in search of a problem scenario’ common in data science). Lastly, but critically, integrating with health systems would require robustness, resilience, stability and flexibility. For example, at least, embedded NLP models should ensure that they are not crashing and/or degrading clinical systems. Such engineering requirements for critical systems are usually not considered and rarely evaluated in the designs and development of research-oriented NLP models.

Albeit these challenges, we observed several exciting translational developments that have been embedded with real-world EHR systems or the near real-time research copies of them. The CogStack^81,140^ text analytics framework has been deployed in more than 5 NHS Trusts across the UK, supporting data harmonisation^144^, semantic search^81^, risk detection and live alerting^145^ and disease prevention^146^. The deployment of text analytics capabilities with health systems has shown its great potential in facilitating more efficient and cost-effective clinical trials^81,147^. Another operational development is the use of clinical NLP models for facilitating efficient medical coding^141^: funded by NIHR recently as an AI Award, University College Hospital colleagues have been comparing^148,149^ for automatically assigning ICD-10 codes for hospital admissions.

The main gap or barrier to clinical NLP in the UK seems to be *impeded research access* to real-world EHR data. First of all, there are no openly accessible free-text EHRs from the UK. All three openly accessible EHRs are from the US. While they are useful for model development and transfer learning (e.g., using pre-trained language models), the significant differences between the US and the UK healthcare systems (for example UK’s discharge summaries are usually much shorter) means that we risk developing models that are less representative of the UK system. Having UK open EHR datasets would allow the community to create benchmarks, train large language models and co-design novel solutions, all of which would greatly speed up the translational processes of research. Secondly, very few TRE are clinical NLP-ready across the UK. The UK now has one of the world’s best TREs (managed by NHS Digital), hosting one of the world’s best national-level health datasets—CVD-COVID-UK https://www.hdruk.ac.uk/projects/cvd-covid-uk-project/. Another notable national initiative is the OpenSAFELY^150^. However, these TREs contain no free-text EHR components at the time of writing. Many local or regional TREs does not support the necessary software environments (e.g., Python or NLP libraries) due to security concerns and/or they do not have the computational resources to support scalable NLP. Thirdly, there are no *shareable* large language models trained on UK EHRs that could facilitate the community for transfer learning. Finally, it is worth mentioning the line of work on synthetic free-text health data generation^151^ for alleviating the pain of data access. Such approaches are in their infancy but could be a promising substitute.

The underlying reason behind the *impeded research access* is perhaps the lack of a streamlined, reproducible and *certified* process for making free-text EHRs research-ready. While there are regulations and guidelines for health data research access, the implementation of these for free-text is very much dependent on the decisions and capacities of local (e.g., NHS Trust level or health board level in Scotland) IG committees, who are frequently overstretched and likely to lack specific experience dealing with free-text health data. A new process of this sort, if adopted, would need to lay out the whole pipeline of data anonymisation and implement the steps from data sampling, preprocessing, annotation, anonymisation, validation, iterative improvements and final reporting. It would ideally be coordinated at a national level and draw on what is a healthily growing area of experience and expertise.

Clinical NLP in the UK is part of a wider international research topic. A full quantitative comparison is outside of the scope of this current review, but we will consider a few points, mainly comparing clinical NLP in the United States (US) with the UK. The majority of clinical NLP is carried out on English language text, with only 10% of NLP papers in PubMed reporting the use of another language^152^. This reflects a broader issue in general NLP, where a small number of languages, first amongst them English, dominate the research literature and the available tools, corpora and representations^153^.

US researchers publish around 6 times the number of AI papers published by UK researchers^154^, and it is reasonable to assume that this is the case for sub-domains such as clinical NLP. This is understandable, given that the US has six times the gross domestic product of the UK^155^ and 5 times the population^156^. Unlike most other national clinical NLP efforts, however, the UK benefits more directly from US research by virtue of the common use of the English language. Despite this, there is a need for specific UK research: terminologies, healthcare systems and clinical cultures all differ.

Compared to the UK, the US has greater levels of clinical NLP in operational healthcare use, as opposed to pure NLP research or epidemiology, this being the result of differing policy pressures. In the US, the Patient Protection and Affordable Care Act 2010^157^, known as Obamacare, and its emphasis on capturing clinical information for meaningful use, has had a direct influence on work to extract as much useful information as possible from EHRs and from patient feedback (see for example^158–160^). In the UK, despite the publication of several white papers encouraging and planning for the use of AI in the NHS (e.g.^161,162^), there has never been a policy impetus as clear as that provided by Obamacare.

There is also a US/UK difference in terms of the available resources, such as clinical corpora and community challenges centred on these corpora. In the US, several corpora are available under lightweight access agreements, most notably MIMIC^143^, but also more specialised corpora such as THYME^163^. Other corpora have been made available for community challenges, such as the series organised by I2B2 (e.g.^164^). The UK’s first semantically annotated corpus of EHR text was reported in^165^. Interestingly, neither the papers reporting this corpus nor the MRC grant that funded it has picked up the searches in the current study. A process was in place for making portions of this UK corpus available to researchers, but it was complex and not used. EHR free text from the CRIS system^166^ is available for use, but under much stricter conditions than the widely-used US corpora. Consequently, there has been a complete absence of UK community challenges, with UK researchers instead participating in US challenges, together with the widespread use of US corpora in UK clinical NLP research.

To close some of the main gaps as a community, the Health Data Research UK’s National Text Analytics Project Consortium (https://www.hdruk.ac.uk/projects/national-text-analytics-project/) has established working groups specifically to create a UK-wide free-text databank and is piloting NLP model sharing of MedCAT models for detecting SNOMED CT concepts with multiple secondary care hospitals in England and internationally including University College Hospital, Kings College Hospital, Guys and St Thomas’, Norfolk and Norwich, Manchester, South London and The Maudsley and University Hospitals Birmingham. The model-sharing agreement and description of community tools can be found on the HDRUK Gateway (https://www.healthdatagateway.org/). To unlock clinical NLP’s full potential for improving health service and patient care, many more initiatives like these are needed with coordination, synergy and collaboration between all stakeholders. In particular, the connections between academia and health service providers need to be expanded and strengthened. Interlinking the UK clinical NLP community with international counterparts is not only nice to have but also essential to address many challenging clinical questions, such as better understanding rare diseases, for which a non-single country could offer sufficient power in their data for revealing evidence. This brings in new challenges, including cross-lingual clinical NLP^167^ and federated NLP^168^. All these gaps and challenges also open exciting opportunities for a better-interlinked community in the UK and beyond.

## Methods

As shown in Fig. 1, the study comprises two parts: one studying the community using network analysis and the other on research and developments using a literature review. While the first is focused on the UK national level, the research outputs include those from the UK as part of international collaborations. This work is not a clinical study, and no personally identifiable information was collected, thus, ethical approval is not required.

### Information collection and data extraction

**Fig. 9:**
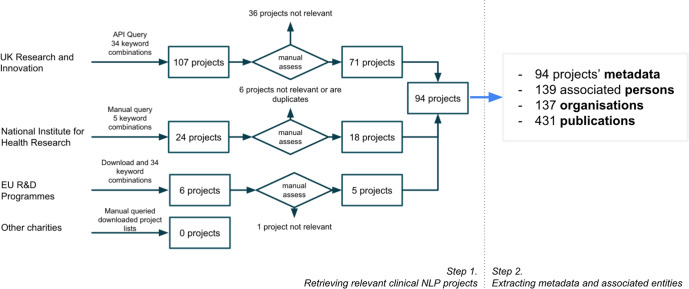
Information collection and data extraction. Step 1: Data were collected for funded clinical NLP projects by querying three searchable datasets from UK and EU funding bodies and downloading project data from UK charities such as British Heart Foundation and Cancer Research UK. Step 2: Data were extracted to obtain metadata of projects and their associated entities.

Figure 9 illustrates our two-step process to (1) retrieve relevant information from online data sources and (2) conduct data extraction to obtain all relevant data for later analysis.

#### Step 1. Retrieving relevant clinical NLP projects

To identify the UK clinical NLP community, we first retrieved relevant projects funded by UK funding bodies (e.g., research councils and charities) and the European Union’s (EU) research and innovation funding programmes. The inclusion criteria were programmes that have (a) developed or applied NLP technologies; (b) solved a clinical, public health or life science research problem that is directly applicable to patient care and (c) involved at least one UK-based organisation.

We started with UK Research and Innovation (UKRI), which “is a non-departmental public body of the Government of the United Kingdom that directs research and innovation funding, funded through the science budget of the Department for Business, Energy and Industrial Strategy". UKRI provides an official Application Programming Interface (API): https://gtr.ukri.org/resources/api.html, which allows efficient access (software-based query and extraction) to successful projects from nine UK-based funders, including seven Research Councils, Innovate UK and Research England^169^. Thirty-four combinations of keyword searches were used to query the web service, which returned 107 unique projects. A manual assessment was then conducted to remove irrelevant projects according to the inclusion criteria, leaving 71 relevant projects.

A similar process was conducted for the UK’s National Institute for Health Research (NIHR), a UK government agency which finances research into health and care. Five keyword searches were used to query the NIHR’s search service (https://fundingawards.nihr.ac.uk/). The NIHR only funds projects for health research allowing us to reduce query combinations from 34 to 5 by using NLP-related keywords only. The search revealed 24 projects, and after a manual assessment, 18 projects were deemed relevant.

For projects funded by European Union’s research programmes, we obtained the data from Horizon 2020-funded projects from https://cordis.europa.eu/projects, which contains all projects from 2014 to January 2022. The same set of UKRI keyword queries was applied to these projects’ meta-data, which identified six projects. After the manual assessment, five were deemed relevant. To enable consistent downstream analysis, the funding amounts of these projects were converted from the original currency (Euro) to Pound Sterling using a rate of 1 to 0.83 (as of 25th January 2022).

Searches of three UK-based charities (Wellcome Trust, Cancer Research UK and British Heart Foundation) did not find relevant projects. Some of these funders do not provide sufficient metadata (e.g., abstracts or summaries) for their funded projects. Therefore, it is possible that relevant projects might have been missed due to incomplete information.

To select projects that fit the inclusion/exclusion criteria, a total of 34 keyword combinations were used. We used broad terms for higher sensitivity followed by a manual second filtering step on query results. The automated retrieval codebase, including the full list of keyword combinations, is available at https://tinyurl.com/5fnvdvrh.

The data collection was finalised on 25th January 2022. Overall, we identified 94 relevant projects. The queries used and extraction scripts are available in a code base referenced at the end of this manuscript.

#### Step 2. Extracting project metadata and associated entities

From the identified projects, we further extended data extraction (see the right part of Fig. 9) to collect project metadata, including title, abstract, technical summary, start/end dates, funding amount, project categories and health categories. For each project, wherever possible, we also extracted its associated entities, including related persons (principal investigators, co-investigators, supervisors/students), organisations (lead organisations, collaborating organisations and their metadata), funders and project outputs (publications, software, datasets and others). In total, from 94 projects, we extracted 139 associated persons, 137 organisations and 431 publications. In particular, for the 137 organisations, we manually classified them into three categories: *research*, *NHS (national health services)* and *industry*.

### Analysis methods

#### Community analysis

To enable an analysis of the UK’s clinical NLP community, we created a network (or interactive graph) linking four types of entities: projects (also called grants), organisations, persons and funders. Links between these entities were directly extracted from the project metadata. The following analysis approaches were conducted.

*(Timestamped and filtered network snapshots)* This analysis reveals the evolution of the community from different perspectives, such as the number of projects, involved persons/organisations and funding budgets over the years and the trend of training the next generation of clinical NLP leaders. The metadata of linked entities (e.g., datetime or project categories) were used to create different snapshots of the network.

*(Centrality analysis)* To identify the ‘key’ stakeholders in the community, centrality analysis^170^ was conducted to quantify node importance in the network. Five centrality measurements were implemented, including degree, betweenness, closeness, eigenvector and PageRank. We report results on eigenvector-based centrality scores (PageRank showed very similar results), which measure the ‘influence’ of nodes in a graph. In particular, we propose a *relative centrality score* metric as an intuitive quantification of node influence among nodes of the same type. It is defined as Eq. (1), where *NodesTypeOf*(*n*) represents the set of nodes that have the same type as *n*. For example, a university with *RCS* = 3 would mean it is very influential in the clinical NLP community—three times more than the median to be exact.

1 $${\rm{RCS}}(n)=\frac{{\rm{centrality}}(n)}{{\rm{median}}(\{{\rm{centrality}}(x)| x\in {\rm{NodesTypeOf}}(n)\})}$$

*(Connectivity analysis)* This is to identify clusters (or components) in a network and quantify the strengths of links between and within different clusters. This allows the identification of the core of the community and, equally important, the weak links among sub-communities. Specifically, we conducted a *k*-connectivity analysis^171^.

*(Force-directed graph visualisation)* This provides an overall representation of the community that enables both inspections of individual entities and illustrates the nature of clusters. Technically, a force-directed visualisation^172^ of the network was implemented to make the network accessible via a browser-based and interactive form.

#### Literature review on research outputs

We conducted a literature review of all publications from the community over the last 15 years to obtain a comprehensive understanding of the research and development of clinical NLP.

*(Information source)* We selected relevant publications from the 431 publications extracted from outputs of the above-mentioned 94 projects.

**Fig. 10:**
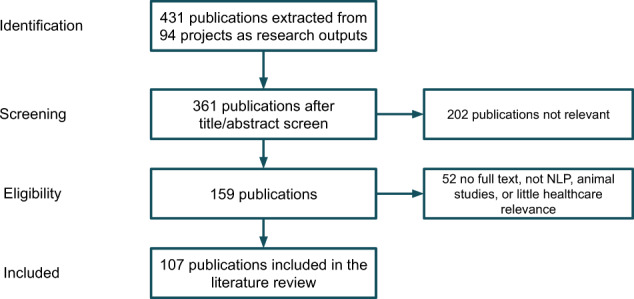
Flow chart describing publication identification for clinical NLP literature review. We started with 431 extracted publications, out of which 361 have sufficient information suitable for screening. The title/abstract screen further removed 202 papers which were deemed irrelevant. This left us 159 publications for an eligibility assessment using inclusion/exclusion criteria on their full text. After this final check step, 107 publications were included for the final review.

*(Eligibility criteria)* The inclusion criteria were: (1) develop or apply NLP technologies; (2) applied in health or life science domains including genetics; (3) full articles including research papers, preprints, conference publications, thesis and book chapters. Exclusion criteria were: (1) animal studies; (2) not full papers (e.g., poster); (3) review articles; (4) articles not accessible. After the screening process, 107 publications were included for final data extraction and review. The Preferred Reporting Items for Systematic Reviews and Meta-Analyses (PRISMA) flowchart of the publication screening and selection process is illustrated in Fig. 10. Two reviewers (J.W. and M.W.) first screened 20 studies independently and achieved full agreement. Thereafter, screening of the remaining studies was performed by the two reviewers independently.

*(Data extraction)* Five reviewers (A.S., F.F., J.W., M.W. and Y.C.) carried out data extraction independently based on a defined protocol. Although there was a risk of bias through independent review, this was reduced by a single reviewer, with MW randomly selecting and double-checking a subset of each reviewer’s results. From these papers, information was extracted on 10 dimensions: (1) publication metadata including title, authors, publication year and article type; (2) international collaborators defined as the countries of co-authors; (3) dataset information including data categories (EHR, social media, literature and others), data source, public availability and data size; (4) health category including disease areas as defined by Clinical Data Interchange Standards Consortium https://www.cdisc.org/standards/therapeutic-areas/disease-area, and disease specification; (5) NLP task types including named entity recognition, entity normalisation, information retrieval, relation extraction, natural language generation, text classification, temporal expression extraction, word sense disambiguation and other information extraction; (6) NLP algorithm category including rule based, ML (not using deep neural network), deep learning, and others; (7) application category as defined in^28^; (8) knowledge representation techniques including ontologies, customised dictionary, pretrained word embeddings, large language models like BERT models^173^ and others; (9) availability of code base and pretrained models; (10) deployment and testing in clinical settings. Missing data was marked as ‘N/A’ during data extraction.

## Data Availability

The data for network analyses and the code for visualising the results are made available at https://observablehq.com/@626e582587f7e383/uk-clinical-nlp-landscaping-analysis.

## Code availability

The code for automatically retrieving projects and their associated metadata is available at https://tinyurl.com/5fnvdvrh.
